# Intensified Local Resource Mobilization for the Polio Eradication Initiative: The Experience of World Health Organization in Nigeria During 2008–2015

**DOI:** 10.1093/infdis/jiv535

**Published:** 2016-04-02

**Authors:** Yared G. Yehualashet, Janet Horton, Pascal Mkanda, Rui G. Vaz, Oluwole Afolabi, Sisay G. Gashu, Richard Banda, Helena O'Malley, Peter Nsubuga

**Affiliations:** 1 World Health Organization, Country Representative Office, Abuja, Nigeria; 2 World Health Organization, Regional Office for Africa, Brazzaville, Congo; 3 Global Public Health Solutions, Atlanta, Georgia

**Keywords:** resource mobilization, donor relations, polio eradication initiative, polio legacy, financial resources requirements, World Health Organization, Nigeria

## Abstract

***Background.*** Since the World Health Assembly (WHA) resolved in 1988 to eradicate poliovirus, several rounds of immunization campaigns have been conducted by member states. By 2000, with the support of the Global Polio Eradication Initiative (GPEI) partners, the number of polio cases decreased by 99%. Eradicating the remaining 1% proved to be more challenging. Although the GPEI, being the largest public health project, required >$9 billion between 1988 and 2012, economic analysis showed the estimated incremental net benefits of $40 billion–$50 billion between 1988 and 2035. In 2012, the WHA declared that the completion of poliovirus eradication is a programmatic emergency for global public health. Nigeria, as one of 3 remaining polio-endemic countries, developed an emergency plan to interrupt the transmission of poliovirus. The plan included the introduction or scale-up of various new innovations and strategies, which had substantial financial implication.

***Methods.*** This is a retrospective study to document the intensified resource mobilization efforts made by the World Health Organization (WHO) in Nigeria to meet the increased financial requirements and bridge the remaining gap in funding. In addition to the established coordination platforms, the WHO Nigeria Country Office team directly engaged with national authorities, donors, and partners throughout the process of resource requirement analysis, project appraisals, proposal development, and implementation of activities, joint monitoring, and evaluation exercises. The office strengthened its capacity for direct funds disbursement and systematic implementation of a rigorous accountability framework.

***Results.*** Between 2008 and May 2015, $538 million was mobilized locally, of which 82% was mobilized since 2012. The percentage of the total funding requirements that were locally mobilized averaged 31% between 2008 and 2011 and increased to 70% between 2012 and May 2015. During the same period, the WHO Nigeria Country Office team produced and submitted 102 grant reports and facilitated >20 joint project assessment exercises.

***Discussion.*** The polio program in Nigeria has achieved unprecedented gains, despite prevailing security and operational challenges, with no case of wild poliovirus infection since July 2014. Through rigorous, transparent, and accountable funds management practice, the WHO country office in Nigeria gained donors' confidence. The locally mobilized funds have made a remarkable contribution to the successful implementation of the strategies set out in the polio emergency plan. We face the challenges of a narrow donor-base, donor fatigue, and competition among other emerging agencies joining the polio eradication initiative efforts over the last few years. We actively engage the national authorities and partners for effective coordination of the polio eradication initiative program and harmonization of resources, using the existing platforms at national, state, and local levels. We recommend strengthening the local resource mobilization machinery and broadening the donor base, to support the polio endgame strategy. Such efforts should also be adopted to support routine immunization, introduction of new vaccines, and strengthening of health systems in the country as part of polio legacy planning.

The World Health Assembly (WHA) resolved in 1988 to eradicate polio from the globe by 2000, as a gift from the 20th century to the 21st century [1]. This assembly also requested immunization partners to support this program, which led to the formation of the Global Polio Eradication Initiative (GPEI). The World Health Organization (WHO), as one of the spearheading partners, was mandated to go beyond its normative and technical role and work alongside governments to implement activities that would lead to the eradication of polio. Since that 1988 resolution that set 2000 as the year to eradicate poliomyelitis, the number of polio cases has fallen by 99% [2]. However, the remaining 1% has proved more challenging to prevent, with the program missing several targets and resetting new ones [3, 4]. Following tremendous effort by national governments and the international community, the world is closer than ever to eradicating polio, with the disease remaining endemic only in Afghanistan, Pakistan, and Nigeria [5–7].

The GPEI is the largest ever public health project and has required billions of dollars to safeguard billions of children from acquiring poliomyelitis. The program operates through intensive grassroots-based operations to administer oral polio vaccines [8]. Despite missing the 2000 target and costing more than $9 billion between 1988 and 2012, polio eradication remained cost-effective, generating net benefits of $27 billion during the same period [9]. Further economic analysis estimated the incremental net benefits of the GPEI to be $40 billion–$50 billion during 1988–2035 [10].

In 2012, the WHA resolved to declare the completion of poliovirus eradication a programmatic emergency for global public health and urged member states with poliovirus transmission to declare such transmission as a national public health emergency [11]. Also at this time, the GPEI released the polio eradication and endgame strategic plan for 2013–2018, which estimated the financial resource requirements (FRR) at $5 billion [12]. In responding to 2012 WHA resolution, the Federal Government of Nigeria (FGoN) declared poliovirus transmission as a national emergency and developed a roadmap to curb the setback it faced with the resurgence of poliovirus transmission in 2011 [13]. With support from the WHO and partners, the FGoN developed the 2012 national polio eradication emergency plan (NPEEP), which contained several innovative strategies to interrupt the transmission as soon as possible [14]. The priority strategies and activities in the 2012 polio eradication initiative (PEI) emergency plan for Nigeria included refining and improving basic strategies, improving performance and motivation of frontline health workers and personnel, scaling up proven innovations, and expanding partnerships and intersectoral collaboration. The plan also included new strategies, such as closer involvement of the country's president through the Presidential Task Force on Polio Eradication, introduction of a national PEI accountability framework at all levels, optimization of new technologies introduced in PEI, systematic introduction of special interventions in security-compromised states, as well as increasing the technical capacity by government and partners in areas at high risk for polio.

The implementation of these new innovations and strategies required substantial financial resources. Subsequently, the Expert Review Committee on Polio Eradication and Routine Immunization in Nigeria, at its meeting in March 2013, called on the FGoN and partners “to move immediately to place the finances of the polio eradication program on firm footing through finalizing existing processes and commitments and pursuing new innovative financing mechanisms” [15].

We describe the experience of the WHO country office in Nigeria in resource mobilization to support PEI activities in Nigeria since 2008 and, in particular, the intensified efforts after the declaration of poliovirus transmission as programmatic emergency at global and national levels in 2012.

## METHODS

We conducted a retrospective review of publications of the global and local PEI partners and working groups, WHO internal documents, secondary sources, and unpublished reports to source data for this article. For the purpose of this article, funds mobilized locally by the WHO country office in Nigeria include domestic contributions from the FGoN that are managed through WHO financial systems and grants from bilateral and multilateral organizations that are negotiated, mobilized, managed, and reported on by the WHO country office.

### Making Resource Mobilization Everyone's Business

To support the intensified PEI activities in 2012, the WHO country office in Nigeria implemented an internal reorganization of staff and procedures. As part of this process, the management revised the terms of reference of the key technical officers and included a component of supporting resource mobilization and donor relations as one of their deliverables.

### Multipartner Coordination

**Figure 1.**
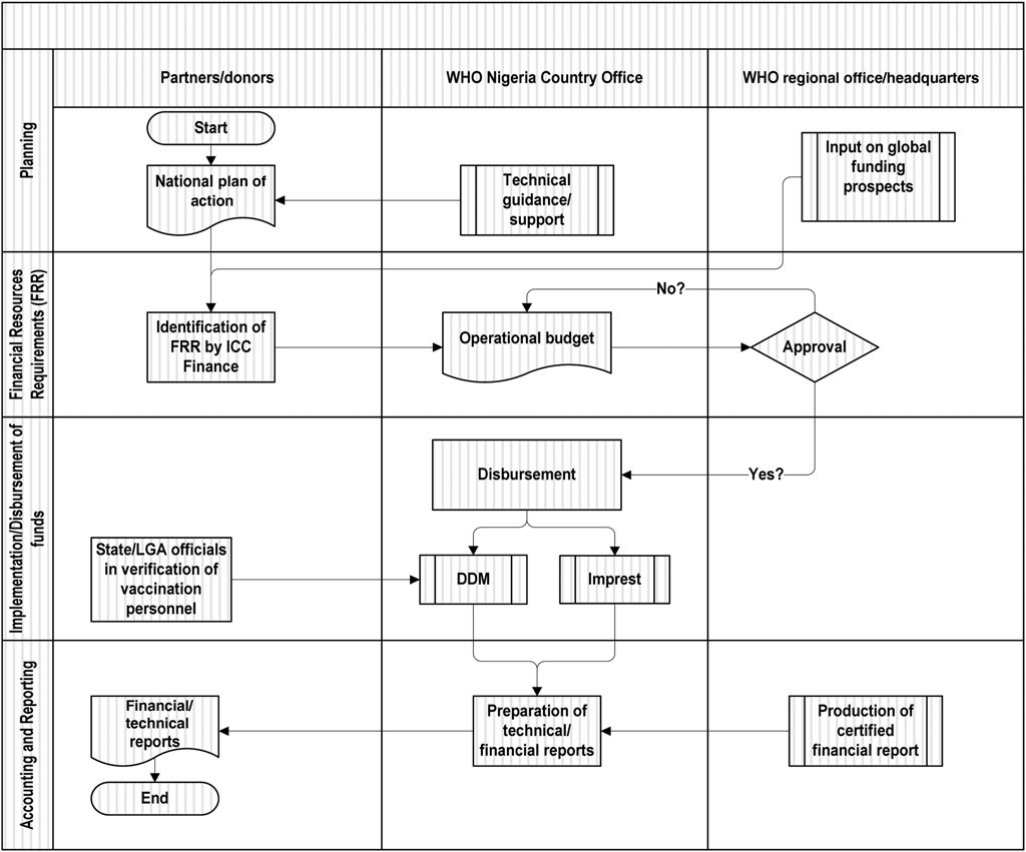
Resource mobilization and management coordination matrix for polio eradication activities in Nigeria. The direct disbursement mechanism (DDM) is a system put in place by the World Health Organization (WHO) in 2004 to disburse funds to the field level for supplementary immunization activities (SIAs) and to provide direct payment to several thousand vaccination personnel engaged at the grassroots level during every SIA round. Imprest is an internal WHO financial system used to electronically produce payment vouchers and financial reconciliations. It is installed at a central location and 37 field offices and is monitored online. Abbreviations: FRR, financial resource requirements; ICC, Inter-Agency Coordinating Committee; LGA, local government authority.

In Nigeria, there are several coordination platforms on health matters at national and local levels. The WHO, as a leading agency, is prominently co-opted into these platforms along with relevant partner agencies. As in the technical sphere, the resource mobilization process is a collaborative effort in Nigeria. Figure 1 demonstrates the high-level WHO coordination mechanism with national authorities and partners in planning the technical requirements, determination of FRR to implement planned activities, deployment of funds, accounting, and reporting on use of the funds. The WHO involves partners in all key aspects of the project management that resonate with the Paris declaration on aid effectiveness [16].

### Identification of the FRR

The FRR provides an overview of the financial requirements for various aspects of the PEI interventions, the funding situation (confirmed and prospective), and funding gaps. In Nigeria, the FRR is developed by the finance subcommittee of the Inter-Agency Coordination Committee (ICC), which is chaired by the FGoN, with the WHO and the United Nations Children's Fund as permanent members. The finance subcommittee regularly compiles inputs from various partners, reviews detailed operational budgets and financial analyses, and produces multiyear FRR (usually covering 3 years). The draft FRR is submitted to the ICC meeting convened by the Federal Minister of Health for endorsement in the presence of heads of major development partners operating in Nigeria for discussion and approval. Once endorsed, the country FRR is submitted to the GPEI secretariat for review and publication. As Table 1 shows, FRR has various components, including requirements for oral polio vaccine, standard operational cost for supplemental immunization activities (SIAs), special interventions in security-compromised areas, polio surveillance, technical assistance, and other in-between activities.

**Table 1.** Major Components of Financial Resource Requirements for Polio Eradication, Main Cost Drivers, and Responsibility for Funds Management in Nigeria, 2015

Component	Percentage of Total 2015 FRR	Main Cost Drivers	Responsibility for Management of Funds Within the FRR Component
Oral polio vaccine	18	Frequency and scope of SIAs, vaccination of target population, and cost of vaccines	UNICEF (100%)
Standard operational costs for SIAs	32	Frequency and scope of SIAs, no. of vaccination teams (as determined by microplanning), and cost of goods and services; major budget lines include vaccination personnel allowances, training and planning, supervision, monitoring and evaluation, intensified SIAs and demand creation, and payment mechanism (all under the WHO) and transport and logistics and social mobilization (both under UNICEF)	WHO (80%) and UNICEF (20%)
Special interventions in security-compromised areas	4	Frequency and scope of interventions, no. of teams engaged, vaccination of target population, and cost of demand creation supplies	WHO (50%) and UNICEF (50%)
Polio surveillance	5	Intensity of field missions for active case-based surveillance, laboratory operations (including transportation of stool samples), and cost of goods and services to maintain 37 field offices	WHO (100%)
Technical assistance	24	Standard UN salary scale and no. of personnel (due to implementation role of WHO and UNICEF) and maintenance of surge capacity personnel since 2012 in states at high risk for polio	WHO (86%) and UNICEF (14%)
In-between activities	17	No. and frequency of activities conducted between regular campaign activities, no. of volunteer community mobilizers engaged, no. of integrated outreach activities in hard-to-reach areas, and no. of routine immunization outreaches in low-performing areas	WHO (22%) and UNICEF (78%)

Contents of the table are the 2015–2017 financial resource requirements endorsed by the Inter-Agency Coordinating Committee on 24 November 2014.

Abbreviations: SIA, supplementary immunization activity; UN, United Nations; UNICEF, United Nations Children's Fund; WHO, World Health Organization.

In addition to the major components of the FRR, the federal and state governments also allocate funds through their own internal systems to support PEI activities at national and state levels.

### Initiating Negotiation With Donors

The WHO country team regularly interacts with national decision makers and country representatives from public and private organizations. These meetings provide the opportunity for discussion on the health issues in the country and current WHO programs, including the PEI. Existing development partners coordination mechanisms also present the platform to initiate discussions on funding prospects. Promising discussions are followed by comprehensive briefings on the status of polio eradication, including strategies developed to address current challenges, as well as operational and program management issues. New donors further conduct project appraisal on the technical, managerial, and operational capability of the organization. The donor mapping document is regularly revised to update the profile of existing and potential donors in the country, to identify possible sources of funding, synergies, and opportunities. The WHO also holds discussions with donor agencies to assess their expectations from the program in general and the WHO in particular.

### Proposal Development

Once the donor is satisfied with the outcome of the project appraisal, they invite the WHO to submit a grant proposal; most often, the donor advises the fund ceiling and the general areas of interest. The WHO generally encourages the donor to allow flexibility in application of the funds within the national polio emergency plan framework and the FRR. This proposal development process involves regular meetings between the program management teams to ensure all technical, legal, and management issues are addressed to the satisfaction of both parties. Among other basic components, a proposal contains grant deliverables such as monitoring indicators and reporting requirements and milestones.

### Agreement Signage

Once the parties involved agree with the terms of the proposal and internal legal and management clearances are obtained, the grant agreement is signed. Some of the agreements require clearance and countersignature from appropriate government agencies. The WHO recognizes the grant in its financial books in preparation for implementation of activities.

### Implementation of Activities Funded

Implementation of activities occurs with joint coordination between the WHO, the national government, and PEI partners. This coordination includes transparent integration of funding requirements and disbursement modalities. Since 2004, the WHO has established a transparent and effective direct disbursement mechanism for timely payment for field-level activities and vaccination personnel allowances. The WHO facilitates donors' participation in field activities to avail them an opportunity to witness the activities supported by their contribution, as well as to give them insight into the opportunities, complexities, and operational challenges the program faces.

### Grant Deliverables

Each grant agreement sets out deliverables to be met within a given period. The frequency and format vary; however, most donors require interim and final financial and technical reports. In some cases, the WHO was required to establish a project steering committee, chaired by the government counterparts, which reviewed annual work plans and project progress periodically. Most grant deliverables are considered timely when submitted within 1 month after the end of the review period. The WHO Nigeria Country Office has a tracking mechanism in place to ensure timely submission of grant deliverables, with the participation of concerned officers and implementation partners.

### Strengthening Donor Relations

Resource mobilization goes beyond raising funds to meet needs; it includes activities such as negotiating for in-kind support or services; cultivating, educating, and servicing donors; and advocating for the cause of the organization (World Health Organization resource mobilization guidelines, 1998, CD ROM). To maximize donor retention, engagement, and investment, the WHO establishes relationships with new donors and nurtures partnerships with current donors by regularly hosting delegations from both outside and within the country and attends functions hosted by donors. These events provide opportunity for the WHO to recognize donor support and express appreciation for their contribution to the polio eradication efforts in the country.

Frequent communication is maintained with the donors through the sharing of weekly polio statistics, monthly epidemiological updates, and relevant articles and reports. This is also supported by regular face-to-face contact with partners at both formal and informal meetings.

### Visibility

To give visibility to donor contribution, we use established forums, such as the GPEI annual reports along with GPEI Polio News [17]. The FRR, published globally, specifies the purpose, amount, and name of the donor that contributed to PEI activities in Nigeria. During field missions, donors have the opportunity for media interviews and to meet senior state-level leaders. The GPEI and WHO Nigeria Country Office websites also host articles on donor field missions. Upon receipt of clearance from the donor, banners carrying the logos of the donor agency are used during selected PEI events.

## RESULTS

### Locally Mobilized Funds

As a result of direct negotiations with donors at the country office level and several grant proposals submitted by the WHO country office, or in line with various memoranda of understanding signed between WHO and the FGoN, $538 million has been mobilized locally and managed through WHO financial systems since 2008. As shown by Figure 1, between 2008 and 2011, local resource contributions averaged $24 million per year. With the intensified resource mobilization activities, the locally mobilized funds markedly increased between 2012 and 2015, during which an average of >$110 million was raised annually.

**Figure 2.**
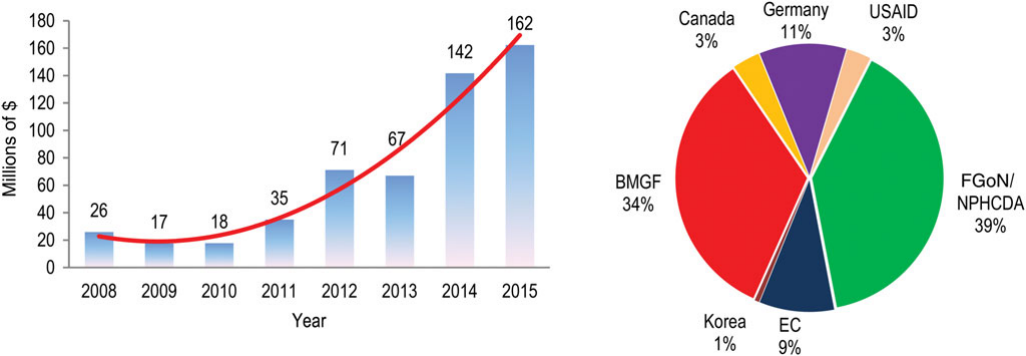
Amount (left) and source (right) of funds mobilized locally to support polio eradication in Nigeria through World Health Organization systems, 2008–May 2015. Data are from World Health Organization internal records and donor agreements. Abbreviations: BMGF, Bill and Melinda Gates Foundation; EC, European Commission; FGoN, Federal Government of Nigeria; NPHCDA, National Primary Health Care Development Agency; USAID, US Agency for International Development.

The proportion of partners' contributions between 2008 and 2015 (May) are shown in Figure 2. Of the total amount mobilized locally, the FGoN contributed 39%, followed by 34% from the Bill and Melinda Gates Foundation, while the remaining partners' contributions accounted for the remaining 27%.

**Figure 3.**
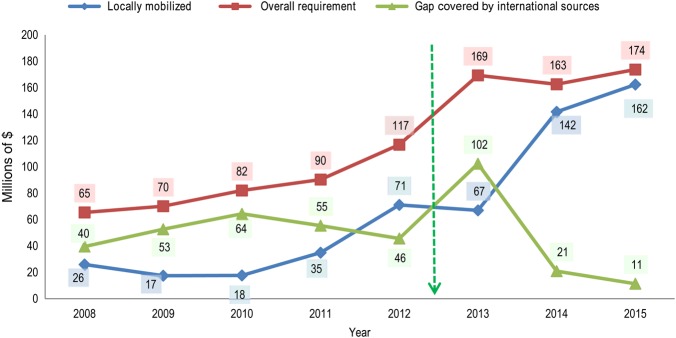
Funds mobilized locally in relation to the overall funding requirement under World Health Organization (WHO)–Nigeria's responsibility and the gap in funding covered by international sources, 2018–May 2015. The green arrow denotes the time at which a number of new innovative strategies were implemented to increase locally mobilized support. Data are from WHO internal records, Global Polio Eradication Initiative financial resources requirements for 2008–2015, and donor agreements.

Figure 3 depicts the local mobilization efforts in relation to the overall funding requirements under the WHO's responsibility and the gaps filled by grants negotiated at the international level during 2008–May 2015. During this period, of the $930 million required to support the PEI budget lines under the WHO's responsibility, 58% ($538 million) was sourced from domestic financing or funds mobilized locally from partners and managed through WHO financial systems, while the remaining gap of 42% ($392 million) was covered by funds mobilized globally through WHO headquarters. The percentage of the total funding requirements that were locally mobilized averaged 31% between 2008 and 2011. A number of new innovative strategies were introduced to effectively the national polio eradication emergency plan. These strategies, which required substantial financial resources, were implemented since mid-2012 (Figure 3). Following the 2012 WHA declaration to intensify PEI efforts and the country office's increased local resource mobilization activities, the percentage of the overall funding requirement satisfied by locally mobilized resources increased to 70% between 2012 and May 2015.

### Submission of Grant Deliverables

The country office generated 102 grant deliverables between 2008 and May 2015. The deliverables included progress and final financial and technical reports, as well as implementation work plans. The number of deliverables increased between 2012 and 2014, averaging 17 reports per annum, compared with 10 deliverables per annum between 2008 and 2011. Besides some challenges the country office faced during late 2011 and early 2012, in the aftermath of the August 2011 bomb attack on the United Nations Abuja office, subsequent donor reports were submitted in a timely manner.

### Project Assessment Missions

In addition to the technical and financial reports submitted to the donors, the WHO country office in Nigeria also organizes joint program assessment missions. Between 2008 and 2014, the office organized >20 joint project appraisal, monitoring, and evaluation activities involving 12 multilateral and bilateral organizations. The activities included 8 field missions that were organized in conjunction with the national and state authorities for delegates of donor agencies to participate in SIAs and surveillance activities, to visit health facilities, and to interact with national and state polio emergency operations centers. Furthermore, meetings that the WHO facilitated with political leaders, partners, and community leaders gave visibility and recognition to the donors' investment by the end beneficiaries. During project appraisal missions, the WHO prepared detailed presentations to update partners on epidemiological, operational, security, and program management issues in the context of PEI in Nigeria.

## DISCUSSION

Nigeria has been making a significant domestic contribution to the PEI [18]. Except for a decrease in 2009, locally mobilized funds have been increasing annually since 2008. The resource mobilization effort was further intensified following the development of the 2012 NPEEP. This effort led to increased donor engagement and a resultant inflow of funds mobilized locally. The implementation of various innovative strategies to increase locally mobilized funds increased the cumulative funding requirements under the WHO's responsibility from $155 million to $506 million between 2013 and 2015. The $371 million mobilized locally by the WHO country office in Nigeria during the same period narrowed the PEI funding gap in the country, thereby alleviating the global resource mobilization burden.

The WHO Nigeria Country Office team also enhanced its capacity to handle the end-to-end grant management processes. Of the 102 reports submitted to donors between 2008 and May 2015, 59% were delivered during the last 3 years. Most donors prefer to use their own grant management instruments, which can be challenging to harmonize with WHO standard policies and practices. However, the country office, in close collaboration with the WHO regional office and headquarters, has been successful in adopting the required flexibility to accommodate donors' varying requirements.

Selling the health agenda in general and PEI in particular is not the most difficult issue. It is rather the ability to guarantee value for money and the judicious and transparent use of resources and systematic implementation of rigorous accountability framework that has enabled the WHO to gain and maintain the confidence of national counterparts and donors to manage such substantial amount of funds.

Although the amount of funds mobilized has increased, the donor-base has narrowed over the years. This posed a risk to critical projects, which required substantial funding as the donors' focus shift to other competing priorities. The challenge was exacerbated by the recent increase in the number of local and international aid agencies that have joined the PEI efforts and become involved in direct field activities instead of supporting the existing implementing partners with funding. There was also enormous pressure to harmonize the ever-increasing donor demands without jeopardizing the values, mandates, and rules of the WHO. Most donors exert a high degree of scrutiny to ascertain value for their investment. Within the provisions of its internal rules and regulations and through established coordination platforms, such as the ICC and emergency operations centers, WHO engaged partners in consultations, planning, monitoring, and evaluation of the program activities and harmonization of resource requirements. In addition to several face-to-face meetings with donors, the WHO organized multiple joint field missions, project appraisals, and verification exercises. These joint exercises were instrumental in building donor confidence in the program in general and in WHO's stewardship in particular.

Prior to 2012, there were occasions when PEI activities had to be postponed or scaled down due to the scarcity of funds from global sources [4, 19, 20]. As Pirlo and Kaufman asserted, “the reality of funding shortfalls undercutting eradication leads to the conclusion that advocacy for resource mobilization is as central to operations as are scientific and technical factors” [19, p. 78].

This article includes neither funds mobilized locally by other PEI partners in Nigeria nor funds managed directly through government systems at national and field levels. However, the WHO has a greater share of responsibility in management of core PEI operational funds in Nigeria. Consequently, the funds mobilized or managed through WHO systems have made substantial contribution in closing the overall funding gap for the program. To manage the risks of donor fatigue, the WHO has used the support of major donors in advocating with high-level government officials and high-profile donors to reinvigorate their financial commitment to the PEI in Nigeria since 2012.

Nigeria has made unprecedented gains in the PEI despite persisting operational and security challenges. The May 2015 independent monitoring board report noted that the country has not had poliovirus since July 2014 and attributed this to the fact that Nigeria has successfully implemented and scaled up the innovations introduced beginning in 2012 [6]. This achievement was made possible through the provision of adequate resources to the program. The substantial funds mobilized locally and the concerted coordination with government and PEI partners at all levels yielded encouraging dividends in this regard.

Government and PEI partners should maintain and even strengthen the local resource mobilization machinery, widen its donor-base, and step up advocacy to ensure that Nigeria successfully implements the polio endgame strategy [12]. As part of polio legacy planning, we also recommend that the local resource mobilization mechanism be applied to support routine immunization, introduce new vaccines, and strengthen health systems in Nigeria.
